# Cervical Spine Osteomyelitis and Epidural Abscess after Chemoradiotherapy for Hypopharyngeal Carcinoma: A Case Report

**DOI:** 10.1155/2014/141307

**Published:** 2014-03-04

**Authors:** Yushi Ueki, Jun Watanabe, Shigehisa Hashimoto, Sugata Takahashi

**Affiliations:** ^1^Otorhonolaryngology Department, Niigata City Genaral Hospital, 463-7 Syumoku, Chuo-Ku, Niigata-shi, Niigata 950-1197, Japan; ^2^Department of Otolaryngology, Head and Neck Surgery, Niigata University, Faculty of Medicine, 757 Ichibancho, Asahimachidori Chuo-ku, Niigata-shi 951-8510, Japan

## Abstract

Osteomyelitis of mandible as a delayed adverse event following radiation therapy has been widely reported; however, osteomyelitis of the cervical spine has rarely been reported. In this study, we reported our experience with a case of cervical spine osteomyelitis and epidural abscess after concurrent chemoradiotherapy (CCRT) for hypopharyngeal carcinoma. The case involved a 68-year old man who underwent radical CCRT after a diagnosis of stage IVb, T4bN2cM0 posterior hypopharyngeal wall carcinoma. At 7 months after completing the initial therapy, the patient complained of severe pain in the neck and both shoulders and reduced muscular strength in the extremities. A large defect was found on the mucosa of posterior hypopharyngeal wall. On cervical magnetic resonance imaging, cervical spine osteomyelitis and an epidural abscess were observed. Because antimicrobial therapy was not effective, hyperbaric oxygen therapy was administered. Abscess reduction and improvement of the mucosal defect were observed. Because cervical spine complications after CCRT can be fatal upon worsening, adequate attention must be given.

## 1. Introduction

Although concurrent chemoradiotherapy (CCRT) is indispensable for the treatment of head and neck carcinomas, various adverse events can occur. Osteonecrosis and osteomyelitis are among these events, and these conditions can worsen after occurrence, leading to potentially fatal results, despite control of the underlying disease. Therefore, these conditions are adverse events that require adequate attention. In this study, we reported our experience with a case of cervical spine osteomyelitis and epidural abscess after CCRT for hypopharyngeal carcinoma with a brief review of the literature.

## 2. Case Report

**Figure 1:**
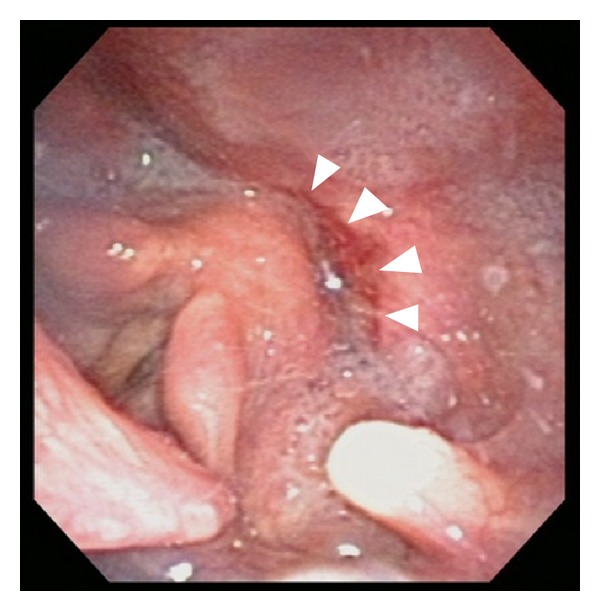
Initial examination findings. Arrowheads indicate a tumor lesion on the posterior wall of the hypopharynx.

**Figure 2:**
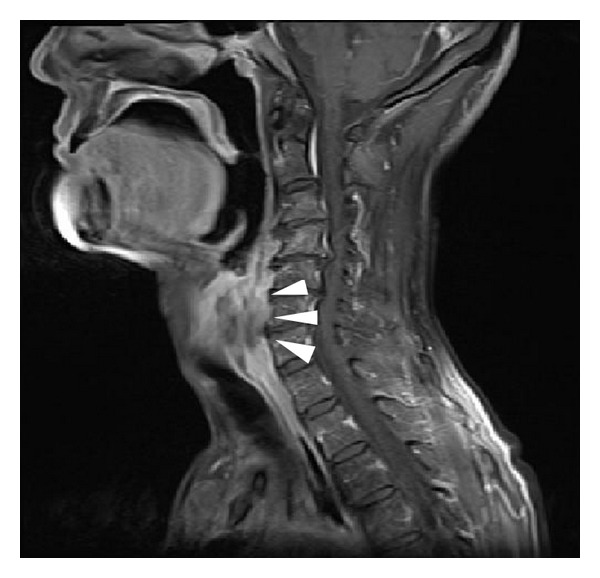
Magnetic resonance T1-weighted, contrast-enhanced image at the first admission. Tumor lesion infiltrated from the posterior wall of hypopharyngeal to the prevertebral muscles.

A 68-year old man complained about swallowing difficulties in December 2011 and visited a nearby clinic in February 2012. He was referred to our department in March 2012 because of a suspected hypopharyngeal tumor. According to the patient's previous medical history, he suffered from hypertension, had smoked 15 cigarettes per day for 50 years, and consumed alcohol at the rate of 3 L of beer per day. During the initial examination, a tumor lesion was found on the posterior wall of the hypopharynx (Figure 1). Enlargement of bilateral retropharyngeal lymph nodes was seen on computed tomography (CT), whereas on magnetic resonance imaging (MRI), lesion infiltration from the posterior wall of hypopharynx to the prevertebral muscles was observed (Figure 2). Squamous cell carcinoma was identified on the basis of biopsy findings, and stage IVb, T4bN2cM0 posterior hypopharyngeal wall carcinoma was diagnosed.

Radical CCRT was performed from April to June 2012. The total radiation therapy dose was 70 Gy, which included a dose of 40 Gy to the whole neck field, followed by an additional 30 Gy to the primary tumor and involved lymph nodes. Cisplatin (70 mg/m^2^) plus 5-fluorouracil (700 mg/m^2^ per day for 5 days) was administered in 2 courses. The lesion disappeared after treatment completion, but the dysphagia did not improve. The patient was discharged after a jejunostomy was established.

After discharge, the patient was followed up and TS-1 was administered as maintenance therapy; however, he experienced a sore throat in November 2012 (5 months after completing CCRT). A recurrence was suspected because visual examination had revealed necrosis of the hypopharyngeal mucosa and incompetence of the vocal cord opening, and thus, the patient was admitted for the second time. Tracheotomy and hypopharyngoscopy revealed extensive necrosis on the posterior wall of the hypopharynx. Pathological examination of the same area did not show the presence of any malignant cells. Because a gas pattern was observed in the prevertebral area on CT images, an infection was suspected. The patient was treated with meropenem (1.5 g/day) for 1 week. He was discharged following improvement in local findings and the sore throat.

**Figure 3:**
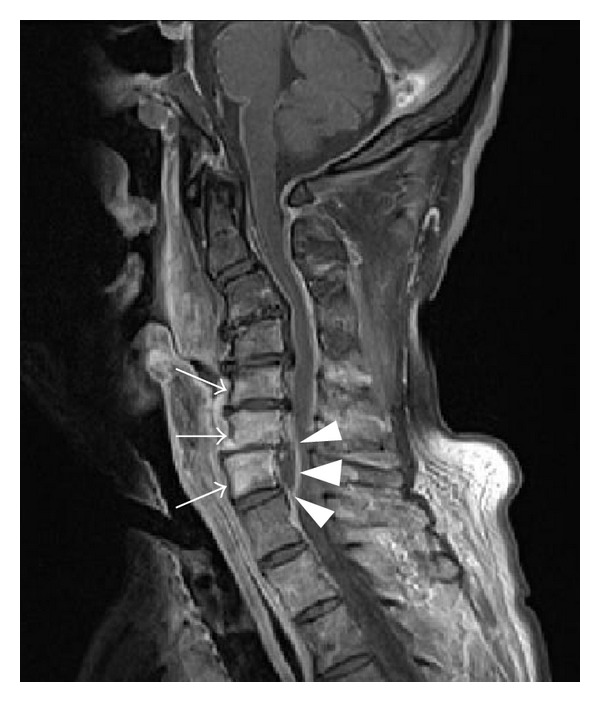
Magnetic resonance T1-weighted, contrast-enhanced image at the time of the third admission. Arrows indicate high signal on vertebrae C4-C7. Arrow heads indicate an epidural abscess.

**Figure 4:**
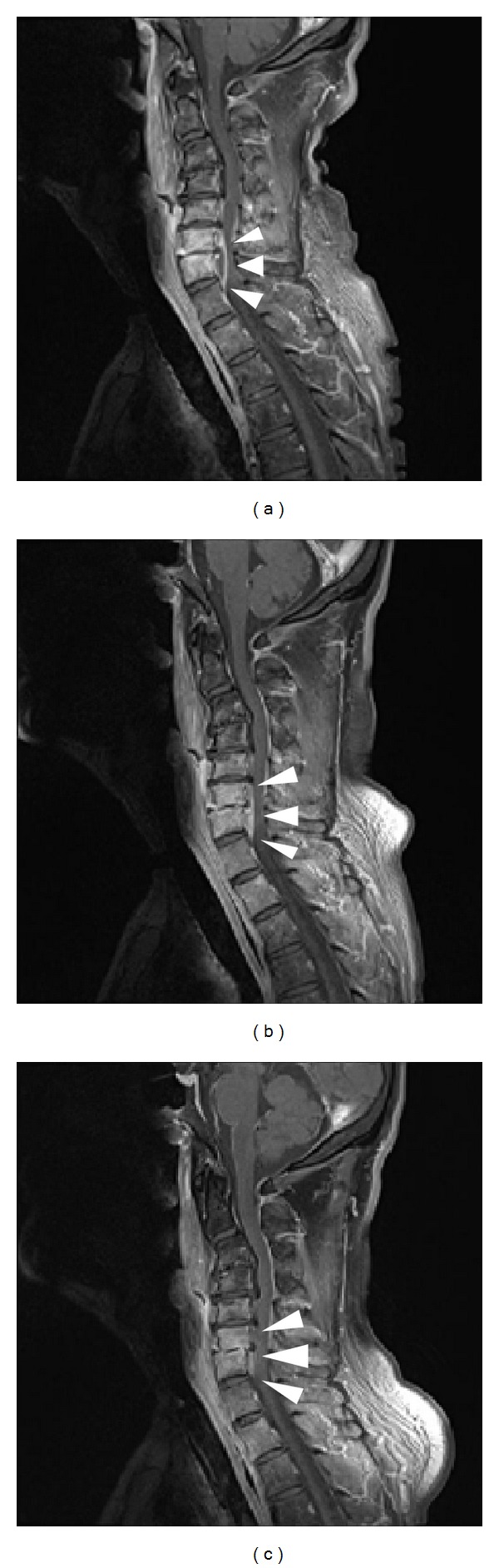
(a) Before initiating hyperbaric oxygen therapy (HBO). (b) After 7 rounds of HBO. (c) After 25 rounds of HBO. The abscess gradually reduced in size and had almost disappeared by the completion of HBO.

In January 2013 (7 months after completing CCRT), the patient experienced pain in both shoulders and the anterior chest, as well as reduced muscle strength in his 4 limbs. He was admitted for the third time for the purpose of close examination. A large mucosal defect was observed on the posterior wall of the hypopharynx. A blood examination revealed a leukocyte count of 10,900/*μ*L and a CRP level of 17.7 mg/dL, suggesting a strong inflammatory reaction; however, no accumulation was observed on bone scintigraphy. Cervical spine osteomyelitis and an epidural abscess on C4-C7 were observed on MRI (Figure 3). The causative bacteria were not identified in a pharyngeal culture. Because the reduced muscle strength worsened despite the administration of meropenem, we discussed treatment for this case with the Orthopedic Surgery Department. We concluded that surgical treatment carried a high risk, and therefore, the patient underwent 25 rounds of hyperbaric oxygen therapy (HBO). The abscess gradually reduced and muscle strength improved, and the patient was subsequently discharged (Figure 4). A defect of the posterior hypopharyngeal wall was also gradually improved. No recurrence has been noted and the patient is followed in outpatient.

## 3. Discussion

Radiation-related osteomyelitis of the mandible has been widely reported, but very few cases of osteomyelitis of the cervical spine have been reported. Prasad et al. [1] reported 84 cases of head and neck osteomyelitis; among these, only 18 cases were of radiation-related osteomyelitis (13 in the mandible, 3 in the maxilla, and only 1 in the cervical spine). Additionally, King et al. [2] reported that, among 884 cases of nasopharyngeal carcinoma treated with radiation therapy, only 9 cases were associated with osteonecrosis in the cervical spine (1%), suggesting that radiation-related necrosis occurred in response to brachytherapy and LASER therapy that were administered as continuation of typical external beam radiation. Kosaka et al. [3] reported 3 cases of cervical spine osteomyelitis caused by radiotherapy for head and neck carcinoma. Accelerated hyperfractionated irradiation or reirradiation was also administered to the same area in these cases.

The standard dose of fractionated irradiation, 70 Gy, was given in this case, but since the patient showed a progressive hypopharyngeal posterior wall carcinoma with infiltration to the prevertebral muscle, it was difficult to reduce the irradiation dose to the cervical spine until the end of the therapy. Additionally, the tumor disappeared after the completion of therapy, although an infection and mucosal deficit occurred in the same area. Because of osteonecrosis of the cervical spine after irradiation and the reduced resistance to infection, healing of the mucosa of posterior hypopharyngeal wall was delayed. Cervical spine osteomyelitis and the epidural abscess were thought to have occurred because of the spreading of infection from the pharynx to the cervical spine. King et al. [2] also indicated the possibility that, because mucosal necrosis occurred at an earlier stage than osteonecrosis, the spreading of infection from this point was involved in osteonecrosis progression. Even in cases such as that described here, which was treated with typical fractionated radiation, cervical spine osteonecrosis can occur as a complication, suggesting that necrosis of the posterior pharyngeal wall mucosa could increase the risk.

Primary lesion necrosis was also observed in the present case, making it difficult to differentiate necrosis from recurrence of the hypopharyngeal carcinoma. In order to differentiate radiation-related osteonecrosis from recurrence, a cervical spine biopsy should be performed, although this procedure is not easily given the inherent risk. It was reported that, in an imaging study of nasopharyngeal carcinoma, the change in MRI intensity from the occipital bone to vertebrae C1-2 and the ulcerative findings of the pharyngeal posterior wall are specific to osteonecrosis [4]; however, there is no remarkable report regarding osteonecrosis of the lower cervical spine. With regard to other modalities, the specificity positron emission tomography in differentiating between osteonecrosis and recurrence was reported that was 85%. However, a false-negative case was also reported, questioning the validity of this argument [5, 6]. Compared to thoracolumbar osteomyelitis, cervical spine osteomyelitis can easily be complicated with comorbid neurological symptoms such as paralysis of the extremities (quadriplegia) and respiratory failure, which are clinical conditions with a high risk of progression. A combination of biopsy and imaging studies should be used to make clinical decisions whenever possible, and early treatment initiation is desirable.

Conservative treatment with antimicrobial agents and surgical therapy are often used for cervical spine osteomyelitis. Initially, antimicrobial therapy was administered in this case, but because of the increased loss in muscle strength of the extremities due to the epidural abscess, we consulted the orthopedic department regarding the possibility of surgical treatment. Adequate debridement and reconstruction in tissues abundant in blood flow are suggested to be effective surgical treatments for radiation-related cervical spine osteonecrosis [7, 8]; however, there is also a high risk of surgical complications such as infection [8]. Since the case was considered a high-risk one for surgical treatment because the patient was at the posttreatment stage of advanced carcinoma and the patient's systemic status was poor (i.e., the patient was malnourished), the patient underwent a noninvasive HBO treatment. HBO is also used to treat radiation-related mandibular osteonecrosis and radiation-related laryngeal necrosis. Although there are no decisive opinions regarding HBO, there have been many reports on its usefulness [9, 10]. We occasionally encounter reports on the use of HBO in addition to surgical treatment or HBO treatment alone for the healing of radiation-related cervical spine osteonecrosis [7, 11]. With regard to surgical treatment, even if the surgical site receives a graft of high blood-flow tissue, because surgery is performed after radiation therapy, there is a high risk of the complications after surgery. As shown here, if the case is a high-risk one for surgical treatment, HBO is thought to be a useful treatment modality for cervical spine osteomyelitis after radiation therapy.

## 4. Conclusion

We experienced a case of cervical spine osteomyelitis and epidural abscess after CCRT for head and neck carcinoma. Cervical spine osteomyelitis after CCRT for head and neck carcinoma is a complication to guard against, since it can lead to a fatal outcome if the condition worsens. Our results suggested that posttreatment necrosis of the posterior pharyngeal wall mucosa can develop into cervical spine osteomyelitis. If the risk associated with surgical treatment is high, HBO is thought to be an effective alternative treatment method.
